# Users’ perception on factors contributing to electronic medical records systems use: a focus group discussion study in healthcare facilities setting in Kenya

**DOI:** 10.1186/s12911-021-01737-x

**Published:** 2021-12-26

**Authors:** Philomena N. Ngugi, Martin C. Were, Ankica Babic

**Affiliations:** 1 Department of Information Science and Media Studies, University of Bergen, Bergen, Norway; 2 Vanderbilt University Medical Center, Nashville, USA

**Keywords:** EMRs use, Facilitators, Barriers, Users’ perception, System users

## Abstract

**Background:**

Electronic medical records systems (EMRs) adoption in healthcare to facilitate work processes have become common in many countries. Although EMRs are associated with quality patient care, patient safety, and cost reduction, their adoption rates are comparatively low. Understanding factors associated with the use of the implemented EMRs are critical for advancing successful implementations and scale-up sustainable initiatives. The aim of this study was to explore end users’ perceptions and experiences on factors facilitating and hindering EMRs use in healthcare facilities in Kenya, a low- and middle-income country.

**Methods:**

Two focus group discussions were conducted with EMRs users (n = 20) each representing a healthcare facility determined by the performance of the EMRs implementation. Content analysis was performed on the transcribed data and relevant themes derived.

**Results:**

Six thematic categories for both facilitators and barriers emerged, and these related to (1) system functionalities; (2) training; (3) technical support; (4) human factors; (5) infrastructure, and (6) EMRs operation mode. The identified facilitators included: easiness of use and learning of the system complemented by EMRs upgrades, efficiency of EMRs in patient data management, responsive information technology (IT) and collegial support, and user training. The identified barriers included: frequent power blackouts, inadequate computers, retrospective data entry EMRs operation mode, lack of continuous training on system upgrades, and delayed IT support.

**Conclusions:**

Users generally believed that the EMRs improved the work process, with multiple factors identified as facilitators and barriers to their use. Most users perceived system functionalities and training as motivators to EMRs use, while infrastructural issues posed as the greatest barrier. No specific EMRs use facilitators and/or barriers could be attributed to facility performance levels. Continuous evaluations are necessary to assess improvements of the identified factors as well as determine emerging issues.

**Supplementary Information:**

The online version contains supplementary material available at 10.1186/s12911-021-01737-x.

## Background

Adoption of electronic medical records systems (EMRs) has been on the rise in both developed and developing countries [1, 2]. EMRs are a repository of patient data in digital form in one practice [3] while electronic health record systems (EHRs) store and securely exchange clinical data, among multiple authorized users beyond one provider’s office [4]. While EMRs adoption rates in developed countries have grown tremendously, in developing countries, the growth rate has been slow and their use is more in administrative rather than for clinical purposes [5]. For example, in the US, EMRs have been in use for over 30 years, with the proportion of hospitals having at least basic EHR functions increasing from 58.9% in 2014 to 80.5% in 2017 [6]. Similarly, Australia has had high rates of EMRs adoption with more than 90% of general practices having some form of EMRs [7]. In New Zealand all general practices use EMRs [8]. On the contrary, studies reveal EMRs adoption has been slow in developing countries due to various factors such as prohibitive costs, security concerns, and interoperability issues among others [9, 10]. However, despite the challenges, many low- to middle-income countries (LMICs) including Ghana, Kenya, Lesotho, Mozambique, Rwanda, Sierra Leone, Tanzania, Uganda, and Zimbabwe have been working on National EMR systems according to a WHO survey [11].

By nature, healthcare organizations are complex and introduction of EMRs can bring further complications, which can lead to rejection of the system or failure of the implementation regardless of the setting [12]. As an example, users are likely to embrace systems that do not interfere with their workflow [13]. Consequently, successful EMRs implementations require careful planning and balancing of service delivery needs in order to optimize the anticipated benefits.

Information system (IS) use is the utilization of information technology (IT) within users’ processes either individually, or within groups or organizations [14]. Implementation of a health information system does not necessarily mean that the system will be used in the way it was intended or used at all. Many factors affect the use of implemented information systems which vary among system users [15]. While these factors may perhaps be generalized regardless of the settings, organizational and user needs can be different due to cultural factors and unique challenges such as inadequate computer to user ratio and power blackouts, among others [9]. Further, the culture of not using information systems for the wider organizational processes (e.g., during health care delivery) may affect their use [16]. Such challenges jeopardize the system’s availability when required for daily use in health care delivery.

While many health professionals generally believe that information technology can eliminate the burden of paper-based documentation such as delays in retrieval of patient records (especially in emergency situations), users get easily discontented if the adopted system or support does not meet their expectations [1, 17, 18]. Further, a study by Myongho revealed that physicians shun systems that interfere with their workflow and how they attend to their patients [19]. Furthermore, several studies have also reported comparatively low adoption rates of EMRs despite broad consensus on the potential benefits such as improved quality of care, patient safety and cost reduction [19–22]. Oftentimes, users hold first-hand knowledge on what can contribute to or limit the use of EMRs implementations as they incorporate these into their work environments [12, 23].

Although there is a corpus of studies conducted to explore barriers and facilitators to EMRs implementations [9, 24–27], only a limited number of evaluation studies are available concerning EMRs use in work processes once the systems are implemented in constrained resource settings [10, 28]. To the best of our knowledge, none of the previous studies have looked at the increasingly deployed national-level EMRs systems in LMICs. Given the significance of users in information system utilization, users’ perceptions are critical in exploring the facilitators and barriers to EMRs use [23]. To this end, this qualitative study set out to identify facilitators and barriers to the nationally-deployed KenyaEMR system (KeEMRs) use in a resource-constrained setting. Facilitors refers to the factors deduced from the participants perspective as motivators to the use of the EMRs in their work process while barriers are factors that hinder use of the system either partially or totally [29]. The goal of this study was to inform EMRs implementations and scale-up strategies in similar settings, considering the high costs involved in such endeavours. This is of interest to various stakeholders including funding agencies and the ministries of health. In this context, ‘use’ refers to a full and/or partial use of the EMRs in all activities relevant to patient care as enabled by the EMRs [14]. This study is part of an ongoing evaluation study on the state of EMRs implementations in Kenya [30].

## Methods

### Study design

A qualitative study design was used to explore perceptions of the users of the electronic medical records system, KeEMRs. The focus was to uncover barriers and facilitators to system use in the healthcare facilities.

### Study global setting for Kenya health system

This study was conducted in Kenya, a country in East Africa with 47 administrative counties and approximately 50 million persons [31]. The Kenyan healthcare system is split into four subsystems namely: Public sector with the major player being Ministry of Health (MoH), Commercial and Non-Governmental Organization (NGO), Private sector, and Faith Based Organisations (FBOs) [32]. The National Health Sector Strategic Plan II introduced the Kenya Essential Package for Health (KEPH), which categorized health service delivery into six levels, which include (1) level 1—community services, (2) level 2—dispensaries/clinics, (3) level 3—health centres, (4) level 4—sub-county hospitals, (5) level 5—county referral hospitals, and (6) level 6—national referrals hospitals [33].

### Study specific setting

In 2012, the Ministry of Health (MoH) in Kenya, with the support of international donor funding and local partners, embarked on development and implementation of EMRs in public healthcare facilities with a view to improve patient data management [34]. Five different types of EMRs developed by separate vendors have been implemented across the country. They include KeEMRs, Ampath Medical Record System (AMRS), eCare, IQCare, and OpenMRS [35]. Nevertheless, KeEMRs and IQCare are the main EMRs accredited to support HIV healthcare delivery services within facilities under the MoH in Kenya [35]. These two EMRs have been rolled out in over 1000 healthcare facilities countrywide [34]. They are mainly deployed in HIV, tuberculosis (TB), and Maternal and Child Health (MCH) clinics, with a view to expanding them to other clinical units in future [36]. The country has since 2019 transitioned to supporting and deploying only KeEMRs. As such, sites running IQCare system are being transitioned to KeEMRs. It is for this reason that this study focused on KeEMRs only.

The healthcare facilities in this study are located in the rural parts of Kenya, spanning eleven counties. They were determined from facilities’ EMRs use performance assessment, conducted in our ongoing evaluation study on the actual use of EMRs implementations in healthcare facilities in Kenya [30]. The assessment utilized computer-generated EMRs *use* empirical data based on various EMRs use indicator measures as outlined in Ngugi et al. [37]. In that study, facility performance was determined by weighted mean calculated from the empirical data of two EMRs use indicators: *Staff System Use* (proportion of active system users) and *Patient Identification* (percentage of patient records with national identification number). From the resulting descending order ranking, facilities were categorized as best performers, average performers and poor performers.

A purposive sample of 20 facilities was selected to provide primary data for this study. Purposive sampling is aimed at seeking depth and richness of information and not representativeness [38]. Drawing from previous studies, a minimum sample size of at least 12 is recommended to reach data saturation for qualitative studies [39, 40]. As such, a sample size of 20 was deemed sufficient for the qualitative analysis and scale of this study as well as accounting for nonresponse. The study facilities were categorized as follows: best performers (top six), average performers (top seven), and poor performers (top seven). Hence, the type of sampling was stratified purposeful sampling. This criteria ensured representation of views and perspectives from facilities at all performance levels. Table 1 presents the characteristics of the 20 healthcare facilities under study.

**Table 1:** Study facilities performance (Top: best, average and poor) and characteristics

Facility performance position*	County no	Weighted mean** (%)	Keph level	Facility_type_category	Owner type	EMRs implementation dates	Services	EMRs mode
1	044	65	Level 2	MEDICAL CLINIC	NGO	12.03.2014	CT&IL	POC
2	042	62	Level 4	HOSPITALS	MoH	01.12.2018	CT	HYBRID
3	039	62	Level 4	HOSPITALS	MoH	27.09.2013	CT	HYBRID
4	042	61	Level 4	HOSPITALS	MoH	01.09.2018	CT&IL	POC
5	042	61	Level 3	HEALTH CENTRE	MoH	01.02.2013	CT	HYBRID
6	029	61	Level 3	HEALTH CENTRE	MoH	04.07.2013	CT	RDE
92	043	41	Level 3	HEALTH CENTRE	MoH	18.09.2014	CT&HTS&IL	HYBRID
93	045	40	Level 4	HOSPITALS	MoH	02.07.2013	CT	RDE
94	037	40	Level 4	HOSPITALS	MoH	25.09.2013	CT	POC
95	018	40	Level 3	HEALTH CENTRE	MoH	26.05.2014	CT	HYBRID
96	045	39	Level 3	HEALTH CENTRE	MoH	24.06.2014	CT	RDE
97	038	39	Level 3	HEALTH CENTRE	MoH	04.08.2014	CT	HYBRID
98	023	39	Level 2	DISPENSARY	FBO	23.07.2013	CT	RDE
207	037	12	Level 3	HEALTH CENTRE	MoH	20.08.2014	CT	HYBRID
208	038	12	Level 3	HEALTH CENTRE	MoH	10.06.2013	CT	HYBRID
209	037	10	Level 3	HEALTH CENTRE	MoH	20.08.2014	CT	HYBRID
210	029	10	Level 3	HOSPITALS	MoH	10.12.2013	CT	RDE
211	038	10	Level 3	HEALTH CENTRE	MoH	17.04.2014	CT	RDE
212	022	9	Level 2	DISPENSARY	FBO	06.11.2013	CT&HTS	HYBRID
213	029	9	Level 4	HOSPITALS	MoH	19.12.2012	CT	POC

*Keph* Kenya essential package for health, *NGO* non governmental organization, *MoH* Ministry of Health, *FBO* Faith Based Organization, CT-Care and Treatment, *HTS* HIV counselling and Testing services, *POC* point of care, *RDE* Retrospective data entry, *IL* interoperability layer

*Positions **1–6**: best performing, **92–98**: average performing and **207–213**: poor performing gauged by **Weighted means of *Staff system use* and *Patient identification* ‘EMRs use’ indicators for the study period 2012–2019. Weighted mean were computed as follows: The two indicators assumed a weighting mean of 1, hence each was assigned a weight of 0.5 in order to have an unbiased mean. A summation of the weighted mean of the mean scores of the two indicators for each facility were then computed and finally ranked in descending order. The two indicators were chosen because they are the key variables that demonstrate EMRs utilization in the healthcare facilities [30]

### The KeEMRs

KeEMRs is an open-source electronic medical records system, that is a customized distribution of OpenMRS developed in Java language [41]. The database (back-end) is developed in MySQL while the user interface (front-end) is developed in JavaScript and hyper text mark up language (HTML). The front-end connects to the back-end via rest application interface (APIs). Since it is open-source software, it uses Linux operating system Ubuntu version 16 and above distribution. OpenMRS is supported by a large global network and used in at least forty countries worldwide [42]. KeEMRs was originally developed by International Training and Education Centre for Health (I-TECH). I-TECH is a global network that works with local partners to develop skilled health care workers and strong national health systems in resource-limited countries [43]. Currently, KeEMRs is supported by Palladium Group through Kenya Management Information System (KHMIS-II) project [44]. Palladium group is an international consulting firm that works in various industries to provide customized solutions [45]. KHMIS-II is a President's Emergency Plan for AIDS Relief (PEPFAR) funded project under the cooperative agreement with Centres for Disease Control and Prevention (CDC) for the period October 2016 to September 2021. The project’s main objective is to support the Ministry of Health, County Health Management teams, and service delivery partners (SDPs) in developing and maintaining health information systems (HIS) innovations in Kenya. There are 31 SDPs in Kenya whose mandate is to deploy EMRs and train users in the healthcare facilities at the county level to support HIV care and treatment. At the time of the study, four of these SDPs were responsible for deploying KeEMRs in the study facilities. Of the trained system users, the facilities selected data staff [mostly health records information officers (HRIOs)] as system champions responsible either at the county or facility level. The selection criteria was based on competency in EMRs usage, enthusiasm, resourcefulness and willingness to learn.

KeEMRs is comprised of different modules to serve various sections of care (majorly HIV and TB clinics) by different categories of users. The main modules include Registration, Triage, HIV testing services, Clinician module, Drug Prescription, Laboratory requests, Patient tracing, Pre-Exposure Prophylaxis service (PrEP), and Reports. The KeEMRs products include: mUzima which is a mobile phone and tablet platform used for HIV testing and counselling (HTS) in offline/online mode, interoperability layers (IL) used for data exchange between systems [e.g. Viral load system from National AIDS and sexually transmitted infections (STI's) control programme (NASCOP) to EMRs], Text messages for adherence (ETS), ARV dispensing tool (ADT), and Data warehouse API (DWAPI)—an application interface that facilitates transmission of HIV indicator data from EMRs to the national data warehouse. An illustration of the national reporting system in Kenya is shown in a supplementary figure (see Additional file 1).

At the time of this study, KeEMRs had been rolled out in over 370 public, non-governmental organizations and faith-based healthcare facilities on varying dates in the period 2012–2018 [43]. The plan of the MoH is to expand the system to all other public facilities and other sections of health care. It is important to note that the implementations of KeEMRs varies in the mode of operation from one healthcare facility to another. The modes include paperless, point of care (POC), retrospective data entry (RDE), and hybrid mode which is a combination of both POC and RDE within the same facility.

### Study participants

A total of 20 participants were recruited from the 20 study sites. Criteria for inclusion in the study was the ability of the participant to inform the conversation around KeEMRs usage, and facilitators and barriers to usage. Thus, the participants were selected by the in-charges of the study sites, as they were well-informed regarding system users suitable to participate in the study. Emphasis was placed on assuring that multiple perspectives were represented in the deliberations. With this in mind, the participants’, who in this study are the units of analysis, included all categories of KeEMRs users including (1) Clinical staff, (2) Nursing staff, (3) Health Records Information Officers (HRIOs), (4) Data entry clerks, and (5) IT staff. Table 2 presents detailed socio-demographic characteristics of the participants.

**Table 2:** Characteristics of the EMRs users participants expressed in frequencies and % (n = 20)

Gender	Male	9(45%)
	Female	11(55%)
Age (years)	20–30	10(50%)
	31–40	10(50%)
Profession	Data clerk	1(5%)
	Health records information officer (HRIO)	15(75%)
	IT staff	2(10%)
	Clinical officer (Clinician)	1(5%)
	Other	1(5%)
Work experience (in years)	< 2	1(5%)
	2–5	10(50%)
	6–10	8(40%)
	> 10	1(5%)
KeEMRs use experience (in years)	< 2	3(15%)
	2–5	13(65%)
	> 5	4(20%)

Four additional participants were drawn from the four SDPs organizations overseeing EMRs implementations in the study facilities. The aim of including this category of participants was to get the management’s perspective, owing to their role in EMRs implementations mentioned earlier. All potential study participants were contacted via telephone and email.

### Data collection

Data were collected through focus group discussions (FGDs) sessions conducted online via a secure Zoom video-conferencing enterprise platform [46, 47]. The choice to use online platform was inevitable due to the social distancing and travel restrictions occasioned by COVID 19 pandemic. However, Reiser et al. [48] purport that online FGDs allows participation across a wide geographical coverage, providing the potential for greater diversified views, among other advantages. Focus group is defined as organized, highly interactive group discussions that aims to explore a specific topic or an issue to generate data [49]. Previous studies have reported varied numbers of participants considered sufficient for an FGD, with the numbers ranging from four to fifteen [50, 51]. Generally, ten participants are considered large enough to gain a variety of perspectives and adequate participation while at the same time small enough to control [52]. We chose this methodology because focus groups are quite suitable for investigating experiences, attitudes and emerging ideas from the group [49]. Additionally, we desired to have an interactive environment where participants would discuss and comment on each other’s experiences and points of view for richness of data (quality).

The 20 participating EMRs users from the study facilities were randomly assigned into two focus groups, each group comprising of 10 participants while the four SDPs representatives formed a third group. There was no specific order of conducting the three FGDs. The group discussions were conducted in English. Each FGD session lasted two hours. The primary researcher (PN-PhD candidate), with FGD training, moderated the FGDs assisted by AB (Associate Professor). The discussions were recorded after explicit permission to record and consent was obtained from each participant. All participants filled a consent form via email before taking part in the study (see Additional file 2).

A list of key questions were used to guide the discussions process (see Additional file 3). The FGDs were initiated by asking all participants to reflect and share briefly on their experiences in supporting care to patients in their respective capacities/roles since the introduction of the KeEMRs in their facilities. This ensured that all participants had a chance to share their views. After this introduction, the moderator’s questions guided the rest of the groups’ discussions. The participants were refunded the cost of internet connection charges incurred to connect to the Zoom platform. All data were collected in July 2020.

### Data analysis

The recorded discussions were downloaded and transcribed verbatim by the researcher, then re-played once to verify accuracy and authenticity. Any participant identifiers or other identifying information were stripped from the data to ensure confidentiality. The anonymized and validated transcripts were then analysed using qualitative content analysis. Content analysis is a systematic analysis of text commonly used in social science [53, 54]. The content analysis process involved coding of the transcribed data followed by categorization into major themes, in line with the questions asked [55]. Through an inductive process, common themes linking codes to categories emerged. Illustrative quotations were abstracted to ground categories, subcategories and themes. The coded data were then categorized as either facilitator or barrier (or both) to EMRs use. Eventually, recommendations towards improvement in EMRs implementation and use were also categorized and summarized. NVivo ver12 qualitative data analysis tool was used to facilitate the data analysis [56]. The study followed consolidated criteria for reporting qualitative studies (COREQ) guidelines. COREQ comprises 32-item checklist developed to promote explicit and comprehensive reporting of interviews and focus groups [57] (see Additional file 4).

The study followed Guba and Lincoln’s criteria of dependability and authenticity [58]. To ensure credibility of the data, the contents of the transcribed data from the focus group discussions were sent to the participants via email to read through and confirm correctness [59]. During the data collection and analysis, the researchers applied reflexivity to avoid biases associated to their own experience on the phenomenon under study by not being actively involved in the discussions (no reactions to participants responses) except guiding the process [60]. To ensure dependability and confirmability, an audit trail was established by keeping a research log of all the activities, developing a data collection record, and clearly recording data analysis procedures. Peer debriefing was also carried out between the FGD sessions and during analysis [58]. Transferability was realized through thick descriptions hence making possible applicability of the findings to other settings implementing EMRs [59].

All methods were carried out in accordance with relevant guidelines and regulations.

## Results

### Facilitators and barriers to EMRs use

**Fig. 1:**
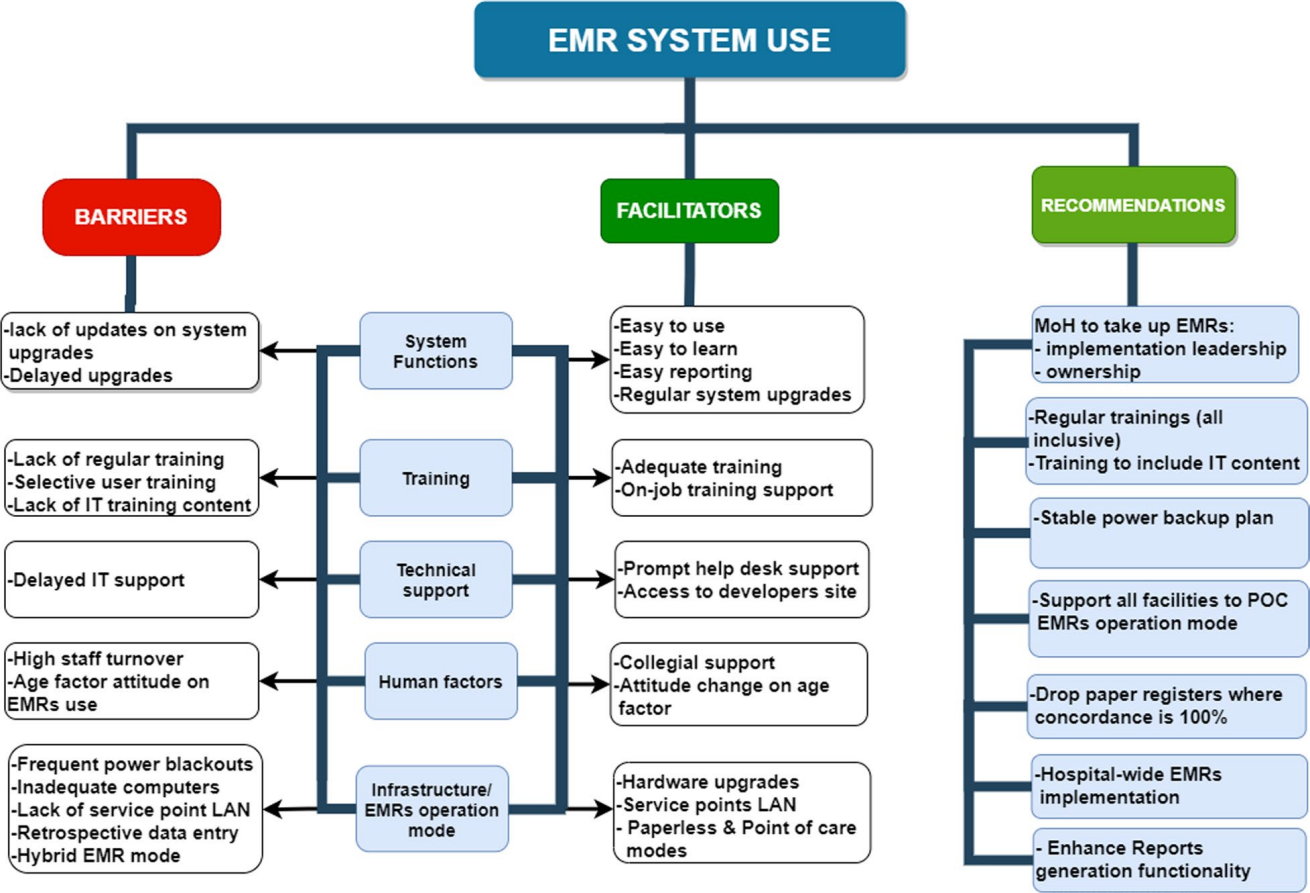
Summary of codes and categories, and recommendations that emerged from the content analysis

From the qualitative analysis of the FGDs data, we identified six categories: (1) System functionalities, (2) training, (3) technical support, (4) EMRs operation mode, (5) human factors, and (6) infrastructure. Results presented below are organized according to the different themes derived from the qualitative content analysis (Fig. 1).

### System functions

Some of the KeEMRs functions include patient registration, patient tracing, ordering laboratory tests, drug prescribing etc. The participants generally believed that KeEMRs is loaded with lots of benefits and it is easy to learn and use. Thus, introduction of the EMRs has been of tremendous help in improving patient care. Before the EMRs implementation in the facilities, patient care was marred with delays and frustrations due to misplaced patient files, wasted time in patient files retrieval and clinicians sieving through the patients’ files thereby constraining time spent with a patient. Staff suffered burn-out occasioning to ‘cooking of data’ in an effort to meet the mandatory monthly reporting requirement to the national MoH aggregate system.

*G1: “……before we started using the system, we had a lot of challenges like patients’ files were missing sometimes. Actually, they were not missing because they were lost, but they were misplaced. So, when a client came, it was very hard to find that file [and as such] the delays were there.”*

*R3: “So after that we started using KenyaEMRs, actually we realised that we are taking a lot of time with the patients, asking patients what is really going on and things are very easy.”*

Further perceived benefits as a result of introducing the EMRs in care delivery include improved patient data management and consequently monthly routine reporting. This has been made possible by the intuitive functions inherent in the system. These includes easy retrieval of patients who have missed appointments, flagging of patients due for viral load, easy way of defaulter tracing and lost to follow-up patients (LFT), management of indicators such as viral loads, and ability to quickly tell current on care and those in differentiated care model. Furthermore, the EMRs users are able to provide prompt and correct information to social department for patient follow-up.

*R7: “Before EMRs, it was difficult to retrieve file if the patient returns after a while—could be after 2 or 3 years. The introduction of EMRs has made it possible to get the patient file by the click of a button (…) we are able to conduct defaulter case management, trace lost to follow-up after they miss clinical visits, pull a list of those patients who have missed appointments, tell the number of days the patients have missed, so we submit the list to the social worker department where they are able to contact the patients so they can come back up to the clinic. The EMR version 17.1.1 is able to give us a list of patients active on care and those who are on differentiated care model.”*

*R1: “Currently what I am appreciating is the management of appointment keeping which we have the calendar in the system which is able to support us as clinical staff. We are able to see how many patients we have booked, what days, so that we do not overbook and under-book on certain days. This has been a very good thing.”*

Additionally, the system users perceived that the EMRs have greatly helped in timely routine reporting as required by both the Ministry of Health (MoH) and service delivery partners (SDPs) based on very reliable data. Facilities are required by the MoH to submit HIV routine data on six programmatic areas based on a summary reporting tool for HIV referred to as MoH731 by the 15th day of every month. The programmatic areas are (1) HIV Counselling and Testing, (2) Care and Treatment, (3) Prevention of Mother to Child Transmission, (4) Voluntary Male Circumcision, (5) Post-Exposure Prophylaxis, and (6) Methadone Assisted Therapy. The reports are sent to the national aggregate system, District Health Information Software Version 2 (DHIS2), maintained by MoH (see Additional file 1). On the other hand, Data for Accountability Transparency and Impact (DATIM) reports are required by supporting partners such as SDPs on quarterly basis.

Most participants indicated that EMRs’ data and paper register records are 100% concordant at the point of care. Concordance refers to the extent to which data contained in paper forms compare to that in the EMRs exuding quality data [61]. Concordance assessment is performed at the facility level by HRIOs. Quality data has gone a long way in supporting decision making at all management levels.

*G4:“Without the EMRs, generating reports like the quarterly and the semi-annual reports could be so tedious. We could spend a whole day in a single facility trying to generate a report for a single quarter. Now our MoH 731 and DATIM reports do not keep changing because we have all these data in place.”*

*G5: “The good thing is we had moved to point of care and our concordance is at 100%.”*

*G1:* “*I want to appreciate the guys working with KenyaEMRs because at [facility name], we have gone very far. We are even sending our reports direct to DHIS2. Actually, we have no problem with reporting (……). We are using DWAPI to send our data to the national data warehouse. We are using the system to send our MoH 731 report direct to DHIS2 whereby nobody can enter it manually it just goes automatically.”*

*G3:* “*With my experience, KenyaEMRs is a good to use. It is really making our work easier and gives us data quality that we can use for decision making at any level.”*

Continuous improvement of the system through regular system upgrades with enhancements of the system’s capabilities was perceived a great motivator in the usage of the system. At the same time, the upgrade process was perceived as non-disruptive.

G2: “*I would like to thank the KenyaEMRs developers for continuous upgrading. We came from version 16.0 ……now we are in version 17.1.1 which has come up with various improvements that were missing from the previous versions. For example, we used to have problems generating the LTF patients which initially included other factors…now all that has been sorted.”*

*U3:“it [upgrades] is done within a few minutes; it is done and then you are able to continue with your work normally.”*

Nevertheless, despite the frequent enhancements of the EMRs as a result of additional new features, the study participants explained that most of the implementing partners delayed in performing the system upgrades at the facilities. Mostly the system enhancements are as a response to users’ demands arising from the need to accomplish their job demands more efficiently through the system. Thus, delays in upgrades may trigger negativity towards the system. This eventually have implications on system use. For example, lack of updates on the EMRs products releases, e.g., the most current—COVID-19 EMR module, was perceived as a usage barrier.

*U6: “The upgrading issue solely lies on the implementing partners, how fast they are.”*

*U3: “…..the modules that were added were not many but this one for COVID 19, I am not sure if it is there because the champions have not been told about it…..”*

### Training

Use of the EMRs in care delivery involves masterly of the system. Thus, users need training to effectively use the system. The study participants perceived the initial training offered at the introduction of the system in facilities, supplemented by on-job-training (OJT) for new untrained staff by colleagues, equipped them with relevant skills necessary to use the EMRs.

*G6: “We had a very thorough training of three days including clinicians, nurses and all the users.”*

*R1: “As much we are getting new staff, the trained ones are able to do OJT to them. Therefore, it has not been difficult for the new staff coming into the facility.”*

While some participants perceived the training they received on how to upgrade the EMRs as a good experience, others had contrary experiences and have to depend on SDPs for the upgrade task. In such situations, the system users lack such skills probably due to either inadequate training or SDPs failure to involve them while conducting such tasks as stated below. As mentioned earlier, SDPs are responsible for system implementation and upgrades tasks at the facilities as well as training.

*U3: “I have been taken through doing the upgrade, and it is a good experience.”*

*G2: “Our implementing partner has done it (upgrade) in the whole [County name]with the new version, it is done within a few minutes and then you are able to continue with your work normally. However, they do not take time to explain to you what has been added.”*

*G1: “…. they (SDPs) just come do the installation or do the upgrades, then they quit and the team who are back there in the facility, they don’t know how to use it.”*

Consequently, many participants expressed the need to have regular trainings in line with the EMRs upgrades as well as refresher sessions. While on-job-training was perceived a good stop gap measure in equipping untrained users, the participants reported that it was not as comprehensive which hamper morale in using the system. Further, the participants explained that it was unclear who should organize for the (re)trainings occasioned by the regular changes in the EMRs implementation structures by the MoH.

*G5: “When the EMR is upgraded, there should be a training for the staffs, all the staffs in the facility. Because you see when it is upgraded some data sets increase and then there is no OJT from the program maybe from [organization name]. So, there should be that regular training to continue giving us morale.”*

*G6: “I don’t know who is to organise all these trainings. Because like right now I can say that almost three years we have never seen something like training but everything is changing.”*

Furthermore, the participants perceived little or lack of IT skills a barrier in executing simple technical or back-end system tasks such as running queries.

G*2: “If something happens, we have to wait for the IT guys to come to our aid on very basic tasks. If at all we had a little background training on the IT, then we can actually perform tasks and make the system up and running and the facility can always continue. So, I think as the champions we should get IT training so that the EMRs can keep on running whether the IT person is around or whether they are not around.”*

Finally, lack of updates by MoH/NASCOP on standard operating procedures, guidelines and definitions such as the period (number days/months) that a patient is considered lost to follow (LTF), which is configured in the system, was identified as a barrier to effective use of the system. As such, the differences in definitions could have effect on decisions made based on erroneous data.

*U7: “KenyaEMR is defining LTF as those who have missed their appointments up to 90 days while in other cases is defined as those missed appointment 29–30 days. These are some of the discrepancies that should be sorted in the system by the supporting partners or NASCOP have to come up with the clear definition of LTF.”*

### Technical support

Another important motivator in the use of the EMRs was the prompt technical support provided at two levels. Level 1 is facility level support where a system error or issue is resolved within the facility by the system champions. If the issue is unresolved it is escalated to the SDPs. However, if the supporting partners are unable to resolve the issue, then it is escalated to KHMIS project level (level 2 support). Level 2 support is provided either through the help desk or by system developers depending on its extent. Level 2 support priority is dependent on the EMRs operation mode where paperless mode is given the highest priority. The participants’ felt prompt support addressed their needs in time and at the same time added to their technical skills.

Further the participants explained access to developer’s website provided information on new system upgrades as well as general support. However, some participants expressed dissatisfaction with IT support within facilities, especially on response time.

*U6: “…..but even before the implementing partners had upgraded, I had already seen there is a package in the KeEMRs website supported by [organization name-EMR developers].”*

*U2: “Actually the challenges that I incur is that there is that error that normally come which takes most of our time. But I want to thank XX from [organization name- EMR developers] who really helped sort that problem. I am also able to sort out some challenges alone without seeking help from [organization name- EMR developers].”*

### Human factors

Participants reported collegial support as a motivator in the use of the system where untrained colleagues learned to use the system through on-job-training (OJT) by colleagues on need basis. The EMRs were initially perceived as a reserve for the young users but through training and teamwork that changed the attitude and now both the young and old are competently using the system.

*R3: “I think everybody in the facility and within the sub-county where I’m working are okay. …I’ve conducted OJT and they are using the EMRs.”*

*G1:* “*So there were bad attitudes towards the system from some of our guys. But actually, after training and having some internal meetings and making sure that the old guys like the facility in charge, she was very old, she was saying she didn’t even know how to touch even the mouse, but actually through the encouragement by colleagues, they have changed … right now, everybody likes the system.”*

Nevertheless, high staff turnover was perceived by most of the participants as a barrier in the use of the system. Those who came in as replacements mostly lacked the capacity to use the system. This was further compounded by the delays in conducting training. Some participants further explained there existed negative attitudes towards the system by staff who are not part of the HIV program hence dismissing it as preserve for specific staff. As such, they were not ready to use the system.

*R7: “The other thing is staff turnover. A staff who is trained on the system could be transferred to another facility. So, we get staff from other facilities who are not trained on the usage of the system. They could be new staff all together.”*

*U5: “As our colleague has said, there has been an attitude that the EMRs is for records personnel only. But I think training should be done not only to the program staffs but should cover all the staff of the facilities…..”*

### Infrastructure and EMRs operation mode

The participants perceived the implementation of the EMRs at all points of care (records, triage, consultation, pharmacy and laboratory) a motivator to system use as it provided seamless flow of patient data in all sections of HIV-care. It also enabled paperless or point of care (POC) EMRs operation modes which meant less work for the system users.

*G1: “We are using KeEMRs in the Comprehensive Care Center (CCC) alone…like in HIV testing and counselling. Counsellors are also using it for testing any person that comes into the facility, even the ‘boda boda’ people (slang for motorcycle riders) and the TB clients. We are also enrolling clients who are HIV positive, even expectant mothers (both positive and non-positive) and children both exposed and non-exposed. It helps us even get the immunization records (….). The system is capturing quite a lot.”*

However, where the EMRs were deployed for use in HIV care only, the participants perceived this a hindrance to its full acceptance and use as they have to revert to paper records while attending to the patients’ other ailments like TB or Malaria*.*

*G2: “However, it is mostly used in the CCC department. So, you find for example, somebody who is at the mother and child health (MCH) clinic will see only CCC clients using the KenyaEMRs and then revert to paper for the other clients.”*

While some participants reported computer hardware upgrades a boost in ensuring system availability, others felt enough was not done to address breakdowns occasioned by hardware failures. Further, lack of enough computers at every point of care was identified a barrier to EMRs use. In such situations, EMRs operate in RDE mode, where patient data is captured on paper and transferred later to the EMRs. Affected users perceived this as cumbersome arising from the double work hence demotivating use. Furthermore, lack of local area network to link service delivery points for seamless data flow as well as care with other clinics within a facility was cited as a barrier.

*R1: “…but recently we have been supported with tablets in the facility, therefore point of care has a tablet where every client (patient) being seen at different delivery points have their details entered in the system.”*

*U3: “….. there are some machines that need to be repaired and some computing part have failed, so I don’t know what is the way forward.”*

*G3: “The only challenge is the replacement of hardware and RAM is slow- speed of the machine. Otherwise, if we can improve on that I am ok. If it can be upgraded am somehow ok.”*

Finally, the most important perceived barrier echoed by participants that cut across nearly all facilities, was lack of appropriate power backup arrangement due to frequent power blackouts. The facilities’ inability to have standby power supply force the users of the system to revert to paper documentation and consequently retrospective data entry. This means double/repeated work, culminating to negative attitude towards system use.

*G3:* “*The main challenge I experience in my facility is power surge and during that power surge we do not have proper backups in our facility so you will have to wait till the power is back …. This finds you when you have even 2–3 days’ work. Network is also an issue at times….I wish we can have power backup so that we do not depend on electricity only.”*

*U6: “We wish we could get a power backup so that we can move from hybrid to POC because as much as I am getting my reports from EMRs, I do that retrospectively, ….”*

*R5: “We don’t have back up, and at the same time we are doing RDE so most of the time we have blackout, like right now we don’t have power so the files are pending until the day power will be back even if it is after one month so it’s really giving us a hard time.”*

## Recommendations for enhanced EMRs use

The study participants provided vital ideas that would address some of the barriers to EMRs use in order to improve its usage. We present these recommendations under the categories of the relevant EMRs implementation stakeholders responsible to act.

### Ministry of health (MoH)

The participants highly recommended that the MoH, being at the national level, take up KeEMRs ownership and lead in its implementations instead of the funding agencies or partners. This will ensure sustainability when the partners leave. Further, MoH should make clear EMRs implementation structure, with the roles of the county governments (hosts of the EMRs implementations), EMRs developers, and SDPs clearly defined. Lastly, the MoH in consultation with NASCOP, should allow facilities with 100% routine data quality assurance (RDQA) concordance to drop some of the paper-based registers to avoid duplication of work, to make EMRs role in routine reporting relevant.

### Service delivery partners (SDPs)

Participants strongly recommended that all facilities be supported in running the EMRs in paperless or POC mode to avoid duplication of work associated with RDE and hybrid modes. Thus, EMRs implementations should be extended to other points of care not just in HIV clinics. Additionally, proper power backup plan (standby generators or solar power) should as well be put in place to address the power blackout challenges, which can last even 2–3 working days halting usage of the system.

All-inclusive training (for both program and MoH staff) was recommended by almost all the participants to eliminate the negative attitude towards the system in terms of who should use the system. To address the user skills gaps in system usage, regular refresher trainings at the facility level as well as system users’ inter-facility forums to share real/practical experiences were recommended. Moreover, the training content should be revised to include basic IT content to enable system champions perform basic back-end staff like running queries at the command prompt.

### KHMIS-II project

While the participants expressed satisfaction with the system functionalities, they did however suggest some improvements especially on reports generation. Currently, the system is designed to generate MoH reports such as MoH 731 (a mandatory summary form for collecting facility HIV-indicator data on monthly basis) by a click of a button. Nevertheless, it is quite cumbersome to generate adhoc reports requested by SDPs using the system. The users have to run a number of other reports to gather data for the requested report. As such, they suggested improvement in reports functionality to support adhoc report generation e.g., through use of queries.

## Discussion and conclusions

This study set out to explore the EMRs users’ perceptions on factors influencing EMRs use in healthcare facilities. Factors related to (1) system functionalities, (2) training, (3) technical support, (4) human factors, (5) infrastructure, and (6) EMRs operation mode were identified as barriers or facilitators to EMRs use. The study findings did not reveal any relationship between the identified factors and the study facilities performance levels.

The EMRs users perceived EMRs implementation in healthcare facilities to have significantly improved patient data management resulting to quick access to patients’ files, high quality data, efficiency in routine reporting, and generally freeing clinician to have more time with the patients. Users perception that EMRs support routine reporting is consistent with Ngugi et al.’s recent study on assessment of HIV data reporting performance by facilities during EMRs implementations in Kenya [36]. Largely, the perception was that all EMRs users were willing to use the system because of the perceived benefits.

EMRs functionalities were perceived adequate in supporting the users perform their tasks. Mostafa et al.’s study in Iran focusing on users’ views and attitudes towards the key elements of successful implementation of hospital information system revealed functional system features as the most important critical success factor [12]. Conversely, systems with missing features and poor performance features are a use barrier [12]. However, variations of clinical functionalities in EMRs implementations is not unusual even in developed countries [62]. Our study demonstrates easy to use and learn qualities of the EMRs coupled with regular system upgrades to address missing functionalities can influence users’ attitude in using the system as posited in other studies [15, 29]. Nonetheless, users reject systems that interfere with the workflow leading to non-use [13].

Several studies emphasize the importance of training as a precursor to information system use regardless of the type [24, 25, 63]. Indeed, the participants in this study elucidated that training gave them the capacity to use the implemented EMRs, which was in some cases conducted informally (on-job training). Nevertheless, the study identified a skills gap among the participants on the capacity to use some functionalities inherent in the system. In fact, some participants were not even aware of existence of certain system functionalities hence a barrier to optimal use. A case in point, some participants indicated they were unaware of COVID 19 EMRs module released to support in the COVID 19 pandemic statistics at the county level. Holden’s study on physicians’ challenges on EMRs use emphasizes unfamiliarity with specific features of a system a barrier to using the EMRs fully, which may possibly be attributed to the quality of training [29]. Further, Pole, in his study of EMRs implementation in Sri Lanka, states that “*the main secret of success was continuous training of hospital staff over a 2 or 3 year period”* [13]. Actually, most of the participants underscored the need for continuous user training especially on the releases of EMRs upgrades. The study also revealed regular trainings that are all inclusive (all staff), can overcome human factor related EMRs use barriers occasioned by high staff turnover and negative attitude.

While results of many studies highlight information system infrastructure challenges such as computers, reliable power supply and networking capabilities, these are however more prevalent in low and medium income countries [9, 27, 64–67], and as well identified in this study. The findings from this study revealed that the challenge of infrastructure did not only affected EMRs availability but also the mode of its operation. Frequent power blackouts resulted into most facilities using the EMRs in RDE or hybrid modes perceived as barriers due to double work (transfer of paper documentation to EMRs when power supply resumes) (see Table 1). The solutions recommended by the study participants including installation of multiple power supplies (e.g. solar, generator, and uninterrupted power supply) and computers at all points of care, are consistent with recommendations by several other studies [28, 65, 66]. Furthermore, since hospital workflows are interconnected where activity in one department (clinic) affects the other, there is need to implement the EMRs in all departments to avoid the undesired shift from EMRs to paper documentation in the process of patient care.

This study identified a gap in the management of the EMRs implementations. The roles of MoH, County government and SDPs around EMRs implementations and management are unclear. This pose a challenge in the implementations leadership and EMRs ownership. For instance, the participants were unsure who should organise for the trainings as well as replace old and slow computers. Further, some facilities were yet to have their EMRs upgraded to latest release. Ismail et al. highlights the importance of high quality project management and detailed planning for successful IS implementations, institutionalization and user acceptance [68].

A key strength of the study was the inclusion of participants from facilities at different performance levels (best, average, and poor) whose characteristics are across-cutting (see Table 1). Further, the participants represented all potential users of the system; clinicians, data clerks, HRIOs and IT staff. This ensured that all possible EMRs implementation and views were well represented. The inclusion of the SDPs in the study also added value in understanding their role in EMRs implementations and in supporting use of the EMRs. The interview guide and appropriate probes ensured that all participants were measured using the same standards (i.e., same environment and use of the same interview guide) [69]. The study FGDs forum made the participants realize the need for similar forums in supporting each other for optimal use of the EMRs features leading to better patient data management.

Nevertheless, there were several limitations to this study. First, due to COVID-19 pandemic, FGDs were conducted using an online modality and therefore we did not collect observational data. Secondly, the two focus groups analysed in this study were the only ones formed and thus the concept of saturation was not applicable [49]. However, enough time was spent within the discussions until there was saturation, with no new information shared [49]. Lastly, the study is limited in generalizability of its findings due to the fact that it was conducted in only one country Kenya, and with one type of EMRs, KeEMRs [70]. Rather, we seek transferability to LMICs contexts similar to this study [59]. Limitations notwithstanding, our study offers crucial important information that will be helpful to decision makers at different levels, including: MoH, funding agencies and local implementing partners for successful EMRs implementations. It is notable that the MoH of Kenya plans to expand the KeEMRs to all other health facilities. Thus, it is expected that the results of this study will shed light on areas that need attention for optimal use of these systems across the country and in similar settings. In particular, power blackout challenges and user training should be given more attention to motivate system usage. Further, the need for clear EMRs implementation structure cannot be over emphasized.

The factors affecting EMRs uptake in resource-constrained settings are complex and need to be better characterized [27] Thus, continuous assessments are also necessary in order to determine improvements and recurrent of similar issues as well, based on previous assessments. This assessment has also complemented other quantitative analyses related to this study [30].

## Supplementary Information

**Additional file 1:** HIV data reporting system in Kenya.

**Additional file 2:** Informed consent form.

**Additional file 3:** Focused group discussion guide.

**Additional file 4:** Consolidated criteria for reporting qualitative studies (COREQ): 32-item checklist.

## Data Availability

The data analyzed in this study is in the custody of the researchers and is available on request. Contact Information: In case of need for the data, please contact: Philomena Ngugi, waruharip@gmail.com.

## Abbreviations

ADT: Antiretroviral dispensing tool
API: Application interface
CCC: Comprehensive care centre
CDC: Centres for disease control and prevention
COREQ: Consolidated criteria for reporting qualitative studies
DATIM: Data for accountability transparency and impact
DHIS2: District health information software version 2
DWAPI: Data warehouse application interface
EHR: Electronic health record
EMR: Electronic medical records
ETS: Text message for adherence
FBOs: Faith based organizations
FGD: Focused group discussion
HRIOs: Health records information officers
HTML: Hyper text mark up language
HTS: HIV testing and counselling
IL: Interoperability layer
IS: Information system
IT: Information technology
KeEMRs: KenyaEMR system
KEPH: Kenya essential package for health
LFT: Lost to follow up
LMIC: Low- and medium-income countries
MCH: Mother and child health
MoH: Ministry of health
NASCOP: National AIDS and STI control programme
NDW: National data warehouse
NGO: Non-governmental organization
OJT: On-job-training
PEPFAR: President's emergency plan for AIDS relief
POC: Point of care
RDE: Retrospective data entry
RDQA: Routine data quality assurance
SDPs: Service delivery partners
STIs: Sexually transmitted infections
TB: Tuberculosis
WHO: World Health Organization
