# Supplementary material for: Users’ perception on factors contributing to electronic medical records systems use: a focus group discussion study in healthcare facilities setting in Kenya
Source: BMC Med Inform Decis Mak. 2021 Dec 26;21:362. doi: 10.1186/s12911-021-01737-x (PMC8710176; doi:10.1186/s12911-021-01737-x)
Supplement: Supplementary file 1 — Additional file 1: HIV data reporting system in Kenya. [file 12911_2021_1737_MOESM1_ESM.pdf]

Additional file 1: HIV Data Reporting System in Kenya

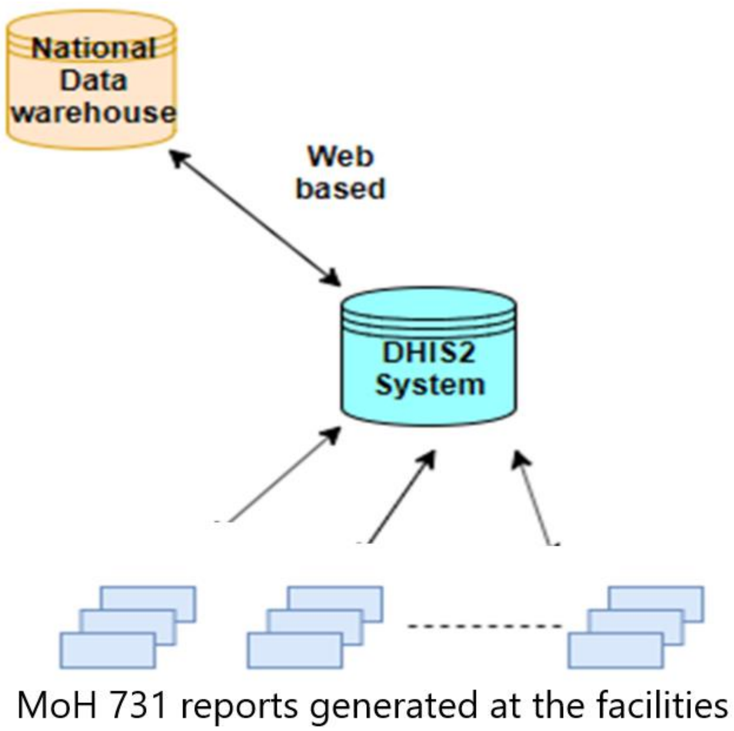

Figure: Ministry of Health (MoH) national reporting system
