# Supplementary material for: Users’ perception on factors contributing to electronic medical records systems use: a focus group discussion study in healthcare facilities setting in Kenya
Source: BMC Med Inform Decis Mak. 2021 Dec 26;21:362. doi: 10.1186/s12911-021-01737-x (PMC8710176; doi:10.1186/s12911-021-01737-x)
Supplement: Supplementary file 2 — Additional file 2: Informed consent form. [file 12911_2021_1737_MOESM2_ESM.pdf]

Additional file 2: Informed Consent Form

<u>Informed consent to participate in a focus group discussion on use, non-use or underuse of EMR systems implementations in healthcare facilities in Kenya.</u>

Protocol Title: Evaluation of the Status of Electronic Medical Records Systems Implementations in Kenya

<u>Purpose of This Research Study</u>

Kenya has embarked on a large-scale roll out of electronic medical records to over 1000 Ministry of Health healthcare facilities. The purpose of this group discussion is to explore end users' perceptions and experiences on factors facilitating and hindering EMRs use in healthcare facilities in Kenya.

You are eligible to take part in this group discussion because your input and experience would be relevant in informing the discussion.

<u>Duration of Participation and Number of People Expected to Participate</u>

<u>Participation Duration:</u> 2 hrs.

<u>Anticipated Number of participants:</u> Approximately 10 people per group.

<u>Description of Procedure</u>

If you agree to participate in the focus group discussion, the following information describes what may be involved.

Due to the social distancing and travel restrictions occasioned by COVID 19 pandemic, the focus group discussions will be conducted online via secure Zoom video-conferencing platform. The link will be shared in due course. The researchers, who have with experience

leading these kinds of discussions, will facilitate the proceedings. The discussions will be recorded and transcribed for analysis.

The perceived facilitators and barriers to EMRs use gathered from the group discussions will be shared to relevant Ministries for action for the success of EMRs implementations countrywide.

Confidentiality of Group Discussion Data

We will not use your name or any other identifying information when we use this information that you are telling us. The only people who will know that you are a participant of the discussion are members of the discussion team. No individual identifying information about you will be disclosed to others, except if required by law. The results of this study will be summarized for reports and may be summarized for presentation at meetings or in publications. No individual identities will be disclosed in any of these reports or presentations.

No personal identifying information will be collected. Discussion records will be stored in locked cabinets in a locked room. Only the evaluation personnel will have access to the data. All computerized information will be protected by access codes known only to the leads of the evaluation and certain designated staff members. All staff members will be trained to keep your information confidential, and they will be informed of the penalty for breach of confidentiality.

Potential Benefits

You may not experience any direct benefit from participating in the focus group discussion. However, a greater understanding about how EMR system usage should be assessed would likely greatly benefit the country by ensuring success of the implementations.

Potential Risks or Discomforts

We will not be collecting any personal health information so there is no risk for loss of private information. Some of the questions in the group discussion may elicit variable opinions and

conflicting responses, which is part of reaching consensus. However, information shared by you will be safely stored and will be confidential, as described above.

Compensation for Internet Connection and Time

Taking part in this group discussion may involve internet connection costs and time. You will be reimbursed for your data buddle and time. The payment will be made at the end of the focus group discussion.

Voluntary Participation and Right to Discontinuation

It is completely voluntary for you to take part in this discussion. If you choose to take part, you can refuse to answer any question or ask us to stop at any time with no penalty. This will not affect your ability to receive any benefits to which you are otherwise entitled.

Disclosure of Financial Interests

Funding for conducting this evaluation is supported by NORHED program (Norad: Project QZA-0484).

Is it okay for you to participate in this focus group discussion via Zoom video-conferencing platform for at most 2 hrs?

YES:______________ NO: ______________

Contact Information:

In case of any questions/complaints regarding this evaluation, please contact:

Philomena Ngugi +254722438086
