# Supplementary material for: Users’ perception on factors contributing to electronic medical records systems use: a focus group discussion study in healthcare facilities setting in Kenya
Source: BMC Med Inform Decis Mak. 2021 Dec 26;21:362. doi: 10.1186/s12911-021-01737-x (PMC8710176; doi:10.1186/s12911-021-01737-x)
Supplement: Supplementary file 3 — Additional file 3: Focused group discussion guide. [file 12911_2021_1737_MOESM3_ESM.pdf]

Additional file 3: Focused Group Discussion Guide.

Introduction

This survey intends to assess the use, underuse, or non- use of EMRs.

Please answer the following questions in the spaces provided, circle or tick the most appropriate options.

1. Age:………………………………………………………………………

2. Gender: (please tick as necessary) □ Male □ Female

3. What is your professional background?

   □ Medical officer /Clinical officer

   □ Nurse

   □ Health Records Information Officer

   □ Data Clerk

   □ IT staff

   □ M&E officer

   □ Other: (please describe) ____________________________________

4. How many years of experience have you had in this current job?

   □ <1 Year □ 1-2 Years

   □ 2-5 Years □ 5-10 Years

   □ >10 Years

5. What is the mode of operation of the Electronic Medical Record System (EMR) in your workplace?

   □ Paperless

   □ Point of care (POC)

   □ Retrospective data entry (RDE)

□ Hybrid (a mix of POC& RDE)

If Hybrid, explain the reason why

____

____

____

____

____

6. How many years have you used Electronic Medical Records System (approximately)?

□ <1 Year □ 1-2 Years

□ 2-5 Years □ 5-10 Years

□ >10 Years

7. Has the EMR been of support in the monthly reporting HIV routine data to DHIS2?

□ Yes □ No

Explain:____

____

____

Thank you for taking the time to complete this questionnaire

FOCUS GROUP: DISCUSSION GUIDE

Facilitator's welcome, introduction, and instructions to participants

Welcome and thank you for volunteering to take part in this focus group. You have been asked to participate as your point of view is important. I realize you are busy and I appreciate your time.

Introduction: This focus group discussion is designed to assess your current experience, thoughts, and feelings about usage of the EMRs implemented in your healthcare facility to

support care delivery, specifically facilitators and barriers in its use. The focus group discussion will take no more than two hours. May I tape the discussion to facilitate its recollection? (If yes, switch on the recorder)

Anonymity: Despite being taped, I would like to assure you that the discussion will be anonymous. The tapes will be kept safely in a locked facility until they are transcribed word for word, then they will be destroyed. The transcribed notes of the focus group will contain no information that would allow individual subjects to be linked to specific statements. You should try to answer and comment as accurately and truthfully as possible. I and the other focus group participants would appreciate it if you would refrain from discussing the comments of other group members outside the focus group. If there are any questions or discussions that you do not wish to answer or participate in, you do not have to do so; however please try to answer and be as involved as possible.

Ground rules

- The most important rule is that only one person speaks at a time. There may be a temptation to jump in when someone is talking but please wait until they have finished.
- There are no right or wrong answers
- You do not have to speak in any particular order
- When you do have something to say, please do so. There are many of you in the group and it is important that I obtain the views of each of you
- You do not have to agree with the views of other people in the group
- Does anyone have any questions? (answers).
- OK, let’s begin

Warm up

- First, I’d like everyone to introduce themselves. Can you tell us your name?

Introductory question

I am just going to give you a couple of minutes to think about your experience of providing care to patients since the introduction EMR (KenyaEMR) system. Is anyone happy to share his or her experience?

Guiding questions

- What are the attitudes of you and other staff towards the EMRs? (What system users' think/say/do?)
- What drove the positive/negative reaction? If negative, how could it be rectified?
- Do you think the EMRs has improved the efficiency of your work? If not, why not? (Similar questions for data quality and routine reporting)
- What are your thoughts on the EMRs functions and capabilities? Are they easy to learn? Do think they cover all the tasks in relation to your role? If not, which ones are lacking?
- What are your thoughts on the training? Did it provide you with the skills you needed to begin using the system effectively? Do you think there is a need for retraining? (if yes, explore which areas and who would need training?)
- When thinking back to how the EMRs was introduced to you, are there ways that could have been introduced to make it easier/better for you?
- What about EMRs upgrades. Which products of KenyaEMR are you aware of? Who does the upgrade? What are your experiences? What are your thoughts on the system upgrade process?
- What are the main issues around actually using the EMRs in your facility?
- What are the barriers to using the EMRs? What are the enablers?
- Did you feel comfortable with using the EMRs? What are some of the issues that hinder your use of the EMRs?
- How would you make it easier to use/implement the EMRs? What are main issues/areas do you think need to be addressed to enhance use of the EMRs?

- Is there anything that we have not covered that you would like to share about your experiences with the EMRs?

Concluding question

- Of all the things we have discussed today, what would you say are the most important issues you would like to express about the EMRs?

Conclusion

- Thank you for participating. This has been a very successful discussion
- Your opinions will be a valuable asset to the study
- We hope you have found the discussion interesting
- If there is anything you are unhappy with or wish to complain about, please contact or speak to me later
- I would like to remind you that any comments featuring in this report will be anonymous
